# Physical working conditions and subsequent disability retirement due to any cause, mental disorders and musculoskeletal diseases: does the risk vary by common mental disorders?

**DOI:** 10.1007/s00127-019-01823-6

**Published:** 2019-12-31

**Authors:** Jaana I. Halonen, Minna Mänty, Olli Pietiläinen, Tero Kujanpää, Noora Kanerva, Jouni Lahti, Eero Lahelma, Ossi Rahkonen, Tea Lallukka

**Affiliations:** 1 Department of Public Health, University of Helsinki, 20, 00014 Helsinki, Finland; 2 Finnish Institute of Occupational Health, Työterveyslaitos, 40, 00032 Helsinki, Finland; 3 Finnish Institute for Health and Welfare, 30, 00271 Helsinki, Finland; 4 Center for Life Course Health Research, Faculty of Medicine, University of Oulu, 8000, 90014 Oulu, Finland

**Keywords:** Common mental disorder, Disability retirement, Physical work

## Abstract

**Purpose:**

Physical work exposures and common mental disorders (CMD) have been linked to increased risk of work disability, but their joint associations with disability retirement due to any cause, mental disorders or musculoskeletal diseases have not been examined.

**Methods:**

The data for exposures and covariates were from the Finnish Helsinki Health Study occupational cohort surveys in 2000–2002, 2007 and 2012. We used 12,458 observations from 6159 employees, who were 40–60 years old at baseline. CMD were measured by the General Health Questionnaire (GHQ-12, cut-off point 3+). Four self-reported work exposures (hazardous exposures, physical workload, computer and shift work) were combined with CMD and categorized as “neither”, “work exposure only”, “CMD only”, and “both”. Associations with register-based disability retirement were assessed with Cox proportional hazards models for sample survey data adjusting for confounders over 5-year follow-up. Additionally, synergy indices were calculated for the combined effects.

**Results:**

Those reporting CMD and high physical workload had a greater risk of disability retirement due to any cause (HR 4.26, 95% CI 3.60–5.03), mental disorders (HR 5.41, 95% CI 3.87–7.56), and musculoskeletal diseases (HR 4.46, 95% CI 3.49–5.71) when compared to those with neither. Synergy indices indicated that these associations were synergistic. Similar associations were observed for CMD and hazardous exposures, but not for combined exposures to CMD and computer or shift work.

**Conclusions:**

Identification of mental health problems among employees in physically demanding jobs is important to support work ability and reduce the risk of premature exit from work due to disability.

**Electronic supplementary material:**

The online version of this article (10.1007/s00127-019-01823-6) contains supplementary material, which is available to authorized users.

## Introduction

Prolonging work careers is increasingly important when the populations are ageing in the developed societies. Early retirement can be a choice of an employee, i.e. conceptualized as retirement as a decision-making, but early retirement due to disability often is an adjustment process that may be related not only to poor health but also to contextual factors leading or adding to poor health [1]. Indeed, good working conditions and health are key factors in keeping individuals at work, and many physical characteristics of work as well as shift work have been linked to an increased risk of early exit such as disability retirement [2–6]. Mental disorders form the second largest diagnosis group for disability retirement after musculoskeletal diseases (MSD) [7]. Even the less severe forms of mental disorders such as self-reported common mental disorders (CMD) have been associated with disability retirement [8–12]. Regardless of the vast evidence on the individual risk factors of disability retirement, it has been less studied if the associations between physical working conditions and disability retirement are shaped by jointly having mental disorders. There is some prior work on the combined effects of psychosocial work characteristics and CMD on work disability [13], but the literature on the combination of physical exposures and CMD on cause-specific disability retirement is, to our knowledge, non-existent.

In this study, we assessed the joint associations of physical work exposures, including shift work, and CMD with the risk of disability retirement. We also assessed synergistic interactions between working conditions and CMD in relation to disability retirement due to any cause as well as due to mental disorders and musculoskeletal diseases. We hypothesized that the associations between physical work exposures and disability retirement would be greater among those who also suffered from CMD when compared to those with neither poor physical working conditions, nor CMD, or only either one.

## Materials and methods

### Study population

We used data from the Helsinki Health Study (HHS), which is a longitudinal cohort study on health and well-being of ageing employees of the City of Helsinki, Finland [14]. The cohort covers a large number of white- and blue-collar occupations from different employment sectors including healthcare, social welfare, education, culture, public transport, and technical services. Our study covered middle-age and ageing employees, among whom ill health and work disability is increasingly common. All the employees of the City of Helsinki, aged 40, 45, 50, 55, and 60 years in 2000, 2001 and 2002 were sent a mailed (asked to participate in) questionnaire gathering information on sociodemographics, health, health behaviours, and working conditions. These age groups were used, based on employees invited to health check-ups by the employer’s occupational health care at 5-year intervals. A total of 8960 employees responded (response rate 67%) at Phase 1 in 2000, 2001 and 2002. Consent was asked for linking the health check-up and survey data; 3815 out of 8960 survey participants participated and consented. However, additional register-based follow-up data for work disability was available for nearly 80% of the baseline survey respondents. The first follow-up, i.e. Phase 2, was collected in 2007 among all respondents of the Phase 1 survey, and the second follow-up, Phase 3, in 2012, again among all baseline respondents, irrespective of their employment status. The survey data have been prospectively linked with the register data from the Finnish Centre for Pensions for all those who gave written informed consent at Phase 1 (74%). Prior non-response analyses have suggested the data are broadly representative of the target population [14, 15], although men, manual workers, and those with long sickness absence have been somewhat overrepresented among the non-respondents.

For this study, we included participants who consented and responded to the survey questions regarding four physical work exposures, CMD and covariates. After excluding those with prior retirement or any missing data (*n* = 151), we included 6159 observations from employed participants at Phase 1, 3839 observations from employed participants at Phase 2, and 2491 observations from employed participants at Phase 3. Thus, the combined data from the three surveys resulted in 12,491 observations from 6159 individuals. Ethics approval was provided by the Ethics Committees of the Department of Public Health, University of Helsinki and the health authorities of the City of Helsinki, Finland.

### Exposures

As exposure variables we used four physical work exposures and CMD. These were identically requested in the three surveys (2000–2002, 2007 and 2012).

#### Physical work exposures

Physical work exposures were assessed based on an 18-item inventory [16]. Using a factor analysis, we obtained three factors of the 18 items, as in prior studies [2, 17]. The first factor comprised items related to hazardous exposures in the work environments including hazardous chemicals, dust, mould, and noise. The second factor comprised items related to heavy physical workload, including uncomfortable postures, repetitive trunk rotation and movements, standing, lifting and carrying. The third factor was called computer work, including items related to working with computer and mouse, and sitting. We divided the loadings for all three factors into quartiles and belonging to the highest quartile indicated exposure to each factor [18]. In all surveys, we also asked whether the participant worked in shifts with six response alternatives. For the analyses, the responses were dichotomized as “normal day work” and “shift work”. Shift work included the following response options: working in day shifts without night work, three-shift work including night work, and night work only.

#### Common mental disorders

As in prior studies, CMD were measured with the General Health Questionnaire (GHQ-12), which has shown comparable results to the original 60-item questionnaire [19]. It has also shown high reliability and good validity in relation to diagnosed affective disorders [19–21]. This measure of CMD includes information on general and context-free affective and non-psychotic problems in the past few weeks, including symptoms of depression, anxiety and poor self-esteem. The GHQ-12 scores range from 0 to 12, and to identify persons with CMD we used a cut-off point 3, which has shown to be a valid threshold for this scale [21]. This threshold is likely to identify employees with CMD.

### Outcomes

Information on work disability retirement was obtained from the Finnish Centre for Pensions that registers all earning-related pensions [22]. In the Finnish system, an earning-related disability pension can be granted to persons aged 18–62 years with a work history that has accrued pension. It is further required that the incapacity to work is estimated to last for at least 1 year. Work disability pension is always assessed by a physician and many factors such as the person’s possibilities of earning a living are considered in the decision, in addition to the diagnosis. This disability pension can be granted either as a temporary, called as fixed-term rehabilitation subsidy, or as a permanent disability pension. Disability retirement (temporary or permanent) for any cause, mental disorder (ICD-10 codes F00–F99) and musculoskeletal disease (M00–M99) were separately examined. These two cause-specific groups were chosen as they form majority of the disability retirements [7]. Follow-up for disability retirement started from the date the survey was returned and continued until the beginning of disability retirement, statutory retirement, death or end of the 5-year follow-up period. This length for the follow-up period was chosen so that those responding to more than one survey would not have overlapping follow-ups. Thus, as new 5-year follow-up period was started after each survey, those responding to all three surveys could have had a maximum of 15 years of follow-up that was stratified in three sections.

### Covariates

Age, gender, marital status, education, body mass index (BMI), smoking status, binge drinking, and somatic diseases were self-reported and considered as covariates as they have shown associations with mental disorders [23–26] and work disability [9, 27–29]. Education was categorized as low = vocational school or less; intermediate = high school or college; high = university degree. Marital statuses were single, married/cohabiting and divorced/widowed. For the calculation of BMI (weight in kilograms divided by height in meters squared), we used weight and height, and it was categorized as non-obese = BMI < 30 kg/m^2^, and obese BMI ≥ 30 kg/m^2^. Smoking was dichotomized into non-smokers vs. current smokers (at the time of the survey). Binge drinking was based on the frequency of having consumed more than six units of alcohol at one occasion, and it was dichotomized as no binging = less than once a month for women and less than once a week for men, and binging = more than once a month for women and once a week for men. For a somatic disease variable, we used information on self-reported physician diagnosed somatic diseases: cardiovascular diseases (angina pectoris, heart attack, cerebral haemorrhage), diabetes, and cancer. This measure was used as a three-class covariate: 0, 1 or ≥ 2 like in a prior study [30].

Average weekly hours of physical activity during leisure time or commuting within the previous 12 months was also requested. We calculated approximate metabolic equivalent (MET) hours per week by multiplying the time spent in physical activity with the MET value of each intensity level and adding these up [31]. For the analyses, we dichotomized weekly MET hours so that < 14 indicated physical inactivity [32]. We considered physical inactivity as a covariate in additional analyses. This was because leisure-time physical activity may buffer the unfavourable effect of physical work exposures on risk of disability retirement [33], suggesting it is a potential mediator in the association between physical work exposures and disability retirement. Additionally, poor mental health has been associated with physical inactivity [34], and vigorous physical activity further with increased risk of disability retirement [35], suggesting mediating role also in the association between CMD and disability retirement.

### Statistical analyses

**Fig. 1:**
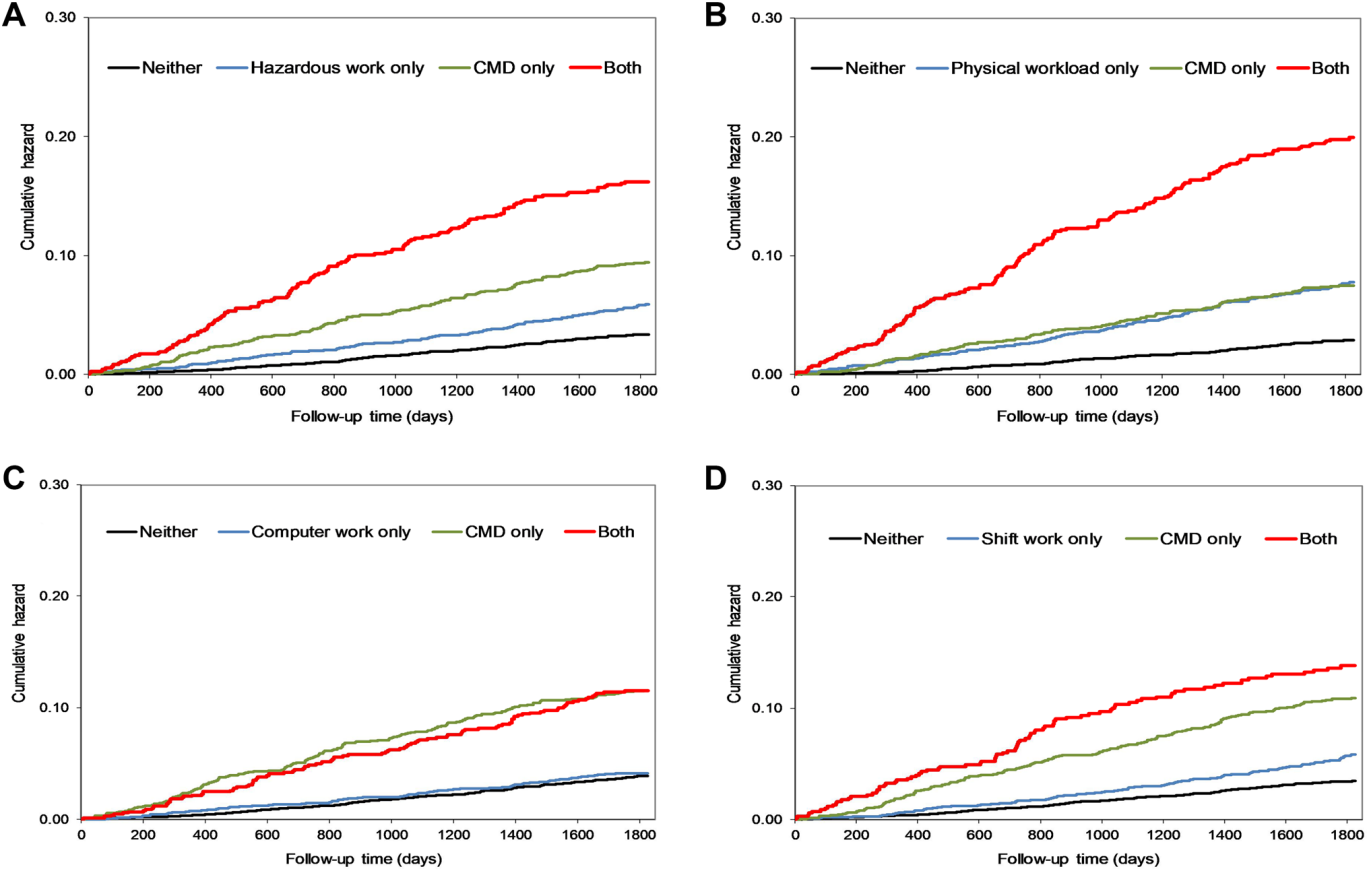
Non-adjusted cumulative hazard of the occurrence of all-cause disability retirement by joint exposure to **a** hazardous work, **b** high physical workload, **c** computer work, **d** shift work and common mental disorders

The 5-year follow-up for disability retirement started from the first day after responding to the questionnaire and ended at the beginning of disability or statutory retirement, death, or end of follow-up (at 5 years), which ever occurred first. For the analyses, we used Cox’s proportional hazards models for sample survey data (proc surveyphreg) that can perform analysis of clustered data, i.e. consider correlations between repeated observations from the same individual. Kaplan–Meier curves (Fig. 1) indicate that the proportionally assumption was fulfilled. We ran two models: first adjusting for gender and age, then additionally adjusting for marital status, education, smoking, binge drinking, BMI and somatic diseases. We observed no gender interactions. In additional analysis, we included physical inactivity in the models. All results are reported as hazard ratios (HR) with their 95% confidence intervals (CI).

In addition, we calculated the synergistic effect for the joint association of work exposures and CMD using synergy indices (*S*, 95% CI) that indicate relative excess risk due to interaction between the work exposures and CMD [36]. Synergy index values above one indicate that the effects of physical work exposure and CMD in combination are greater than one would expect from these factors in isolation [37]. We used the following formula for calculating synergy indices for each set of two dichotomised variables: *S* = (RRA+B+ − 1)/[(RRA+B− − 1) + (RRA-B+ − 1)] [38]. Here RRA+B+ is the relative risk of disability retirement if both factors A (physical work exposure) and B (CMD) are present, RRA+B− is the relative risk of disability retirement if A is present but B is absent, and RRA-B+ is the relative risk of disability retirement if B is present but A is absent.

## Results

Descriptive statistics of the categorized variables for the total study population, and by CMD status are presented in Table 1. Most (80%) of the study participants were women, mean age at the start of the follow-up for disability retirement was 51.8 (SD = 6.3) years, and mean score for GHQ-12 was 1.9 (SD = 3.1). Of all participants 24% were physically inactive, 31% of those with CMD and 22% of those without CMD. Numbers of disability retirements by the four exposure variables are shown in Table 2. Mean follow-up time in the three 5-year strata was 4.2 (SD = 1.4) years.

**Table 1:** Descriptive data on the study population and by common mental disorders (CMD) at baseline

Variables	All (*n* = 6159)		CMD (*n* = 1504)		No CMD (*n* = 4655)	
	*n*	%	*n*	%	*n*	%
Social factors						
Women	4826	78	1193	79	3633	78
Marital status						
Single	766	12	197	13	569	12
Married/cohabiting	4355	71	1020	68	3335	72
Divorced/widowed	1038	17	287	19	751	16
Education						
Low	2424	39	572	38	1852	40
Intermediate	2016	33	502	33	1514	32
High	1719	28	430	29	1289	28
Health-related factors						
Smoker	1416	23	396	26	1020	22
Binge drinker	2924	47	774	51	2150	46
Obese	896	15	247	16	659	14
Somatic disease						
0	5416	88	1292	86	4124	88
1	687	11	194	13	493	11
≥ 2	56	1	18	1	38	1
Physical working conditions						
Hazardous exposures	1508	25	461	31	1047	22
Physical workload	1499	24	487	32	1012	22
Computer work	1543	25	511	34	1032	22
Shift work	1404	23	357	24	1047	22

**Table 2:** Number of work disability retirements by cause and by the exposure variables within the 5-year follow-up

Exposure	Number of observations	Any cause	Mental disorder	Musculoskeletal disease
Hazardous exposures/CMD				
Neither	7379	523	117	235
Work exposure only	2145	248	45	141
CMD only	2053	296	121	94
Both	914	228	67	101
Physical workload/CMD				
Neither	7462	475	120	197
Work exposure only	2062	296	42	179
CMD only	2014	242	109	59
Both	953	282	79	136
Computer work/CMD				
Neither	7403	597	108	309
Work exposure only	2121	174	54	67
CMD only	1943	358	118	146
Both	1024	166	70	49
Shift work/CMD				
Neither	7561	537	115	259
Work exposure only	1963	234	47	117
CMD only	2299	382	148	135
Both	668	142	40	60

Figure 1 shows the unadjusted cumulative hazard of the occurrence of work disability due to any cause by the four work exposures and CMD. Corresponding figures for disability retirement due to mental disorders and musculoskeletal diseases are shown in Supplemental Figs. 1 and 2.

In Supplemental Table 1, we present age- and gender-adjusted HRs for disability retirement due to any cause, mental disorders, and musculoskeletal diseases by the four exposure variables, as well as the synergy indices. All combined exposures except that for computer work and CMD resulted in the highest hazards for disability retirement. Synergy indices for physical workload and CMD were above one and statistically significant for all three outcome measures, suggesting larger effects for the joint exposure than what would be expected based on individually assessed estimates. Hazardous exposures and CMD indicated synergism in relation to disability retirement due to any cause (*S* = 1.63, 95% CI 1.40–1.97) and musculoskeletal diseases (*S* = 1.79, 95% CI 1.19–2.70).

Fully adjusted estimates for any disability retirement are provided in Supplemental Table 2. Of the work exposures alone, physical workload was most strongly associated with any disability retirement after full adjustments (HR 1.89, 95% CI 1.61–2.23), whereas the association for computer work was weaker and non-significant. However, the associations between CMD alone and any disability retirement (HRs ranging between 1.95 and 2.43) were stronger than the associations for the work exposures alone. The joint exposure to hazardous exposures and CMD (HR 3.20, 95% CI 2.70–3.80) and physical workload and CMD (HR 4.26, 95% CI 3.60–5.03) had the strongest associations with any disability retirement.

**Fig. 2:**
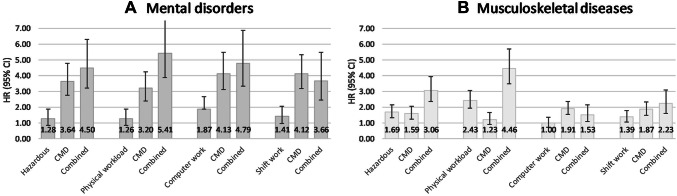
Fully adjusted hazard ratios (HR, 95% confidence intervals) for disability retirement due to **a** mental disorders and **b** musculoskeletal diseases by categories of the four exposure variables, “neither” group as the reference

Fully adjusted estimates for disability retirement for mental disorders are shown in Fig. 2 panel A. Computer work was the strongest work-related predictor of disability retirement due to mental disorders. However, CMD alone was more strongly associated with disability retirement due to mental disorders than any of the work exposures alone. Of the combined exposures, the strongest association was observed for physical workload and CMD (HR 5.41, 95% CI 3.87–7.56), for which the synergy index also remained significant (*S* = 1.80, 95% CI 1.18–2.72).

Fully adjusted estimates for disability retirement due to musculoskeletal diseases are shown in Fig. 2 panel B. Physical workload alone had the strongest association with disability retirement due to musculoskeletal diseases (HR 2.43, 95% CI 1.93–3.07), and this association was stronger than that for CMD alone. Of the combined exposures, the strongest associations with disability retirement due to musculoskeletal diseases were observed for physical workload and CMD (HR 4.46, 95% CI 3.49–5.71) and hazardous exposures and CMD (HR 3.06, 95% CI 2.37–3.95), and these were significantly stronger than associations for the exposures alone. The synergy indices also remained statistically significant for these exposures: *S* = 2.09 (95% CI 1.43–3.06) for physical workload and CMD, and *S* = 1.60 (95% 1.01–2.54) for hazardous exposures and CMD. Additional adjustment for physical inactivity generally slightly strengthened the associations between the combined exposures to physical work and CMD and disability retirement (Supplemental Table 3).

## Discussion

We observed that the risk of disability retirement due to any cause, mental disorders or musculoskeletal diseases was greater among those with combined exposure to high physical workload and CMD or with hazardous exposures and CMD when compared to those with neither or only one exposure. Importantly, these factors interacted so that their joint effects on disability retirement were greater than what would be expected from these factors if assessed individually. Associations for the combined exposures to computer or shift work and CMD with disability retirement did not markedly differ from those for CMD alone.

In line with the current findings, there is evidence that physically heavy work alone increases the risk of disability retirement due to any cause [39]. Looking at the causes of disability retirement more closely, others have shown that heavy physical work increased the risk of disability retirement due to musculoskeletal disorders in particular [2, 4, 5, 40], which agrees with our results. In the earlier studies, however, the additional burden of mental disorders has not been examined in detail. The observed synergy between the two examined physical work exposures, i.e. high physical workload and hazardous exposures, and CMD in relation to disability retirement not only due to musculoskeletal diseases, but also due to mental disorders is a noteworthy finding. It suggests that it is important to identify those employees whose work includes these physical work exposures and who also suffer from common mental disorders, as their combined effects on work ability may be particularly harmful. These variables can be viewed as background variables that shape the decision-making related to early retirement.

When further looking at disability retirement due to mental disorders, CMD alone was a strong predictor, as observed in prior studies with no linkage to physical work [8, 11]. Hazard of disability retirement due to mental disorders, but not musculoskeletal diseases, was increased by exposure to computer work alone, although the association with CMD alone was much stronger. Shift work alone was weakly associated with all examined outcomes, although the effect estimate for disability retirement due to mental disorders did not reach statistical significance in the fully adjusted model. The existing evidence for the association between shift work and disability retirement in general is mixed [41], but a recent study reported an association between night work and an increased risk of disability retirement due to musculoskeletal diseases [42].

The major strengths of this study are the large employee cohort including a broad range of different occupations, the longitudinal study design, and reliable register data on cause-specific disability retirements. Physical work exposures were broadly assessed based on an 18-item question battery and information of shift work that are major risk factors of work disability. In addition, although the exposures were subjectively assessed, the information was obtained for each individual whereas some prior studies have assessed cumulative exposures at group level based on job titles [43]. However, some limitations need to be considered. The use of self-reported measures for the work exposures and CMD may have caused reporting bias and either under- or overestimation of the actual exposures. For example, it is possible that individuals with CMD are biased towards reporting higher levels of physical exposures at work. One mechanism for this may be through the personality trait neuroticism which seems to be associated with a tendency for negative effect, symptoms, and over-reporting of adverse exposures. We defined CMD using the GHQ-12 questionnaire that covers symptoms of minor mental health problems. This measure does not distinguish between less and more severe conditions or depression and anxiety, but it has been shown to be a reliable and valid measure suitable for use in general and employee populations [44]. These findings may also be affected by non-response and the healthy worker effect that might lead to underestimation of the findings, although previous non-response analysis has shown that these data represent well the target population [14, 15]. The mean age at the beginning of follow-up for disability retirement was 51.8 years, which may have led to conservative effect estimates if compared to study samples consisting of older employees. However, we consider these findings relevant as the actions to decrease exposure to adverse physical working conditions might be especially important for the younger employees who will have longer careers ahead of them. As the data included employees only from one employer within the Finnish municipal sector, the generalizability of our findings to the public sector in general, private sector, or to employed populations in other countries may be limited. However, while there are between-context differences in, for example, factors related to work legislation, social security, access to healthcare and macroeconomic conditions, there is no specific reason to assume that the observed associations would remarkably differ between workplaces.

## Conclusions

We observed synergistic interactions between exposure to hazardous exposures or high physical workload and CMD in relation to disability retirement due to any cause, mental disorders and musculoskeletal diseases. These findings underline the vulnerability of, and highlight the importance of, recognizing those employees who have physically heavy work tasks and simultaneously suffer from common mental disorders. For them, actions to alleviate demanding work conditions and to provide support in mental health issues should be planned in collaboration with employees, employers and (occupational) health professionals.

## Electronic supplementary material

Below is the link to the electronic supplementary material.

Supplementary material 1 (DOCX 520 kb)
