# Supplementary material for: Physical working conditions and subsequent disability retirement due to any cause, mental disorders and musculoskeletal diseases: does the risk vary by common mental disorders?
Source: Soc Psychiatry Psychiatr Epidemiol. 2019 Dec 31;55(8):1021–9. doi: 10.1007/s00127-019-01823-6 (PMC7395011; doi:10.1007/s00127-019-01823-6)
Supplement: Supplementary file 1 — Supplementary material 1 (DOCX 520 kb) [file 127_2019_1823_MOESM1_ESM.docx]

**Supplemental material - Social Psychiatry and Psychiatric Epidemiology**

Physical working conditions and subsequent disability retirement due to any cause, mental disorders and musculoskeletal diseases: does the risk vary by common mental disorders?

Jaana I. Halonen,^1,2^ Minna Mänty,^1^ Olli Pietiläinen,^1^ Tero Kujanpää,^3^ Noora Kanerva,^1^ Jouni Lahti,^1^ Eero Lahelma,^1^ Ossi Rahkonen,^1^ Tea Lallukka^1,2^

1) Department of Public Health, University of Helsinki, P.O. Box 20, 00014 University of Helsinki, Finland

2) Finnish Institute of Occupational Health, Helsinki, P.O. Box 40, 00032 Työterveyslaitos, Finland

3) Center for Life Course Health Research, Faculty of Medicine, University of Oulu, P.O. Box 8000, 90014 University of Oulu, Finland

Corresponding author: Dr. Jaana I. Halonen,

Current address: Finnish Institute of Occupational Health, 70032 TYÖTERVEYSLAITOS, Finland

E-mail: [jaana.halonen@ttl.fi](mailto:jaana.halonen@ttl.fi)

Telephone: +358 43 82 44 264

**Supplemental Table 1.** Age- and gender-adjusted hazard ratios (HR, 95% confidence intervals) and synergy indexes (S, 95% confidence interval) for disability retirement by categories of the exposure variables.

|  | | Any cause | | | | Mental disorders | | | | Musculoskeletal diseases | | | | | | |
| --- | --- | --- | --- | --- | --- | --- | --- | --- | --- | --- | --- | --- | --- | --- | --- | --- |
| **Exposure** | HR | | 95% CI | | HR | | 95% CI | | HR | | | 95% CI | | | |  |
| Hazardous exposures/ CMD |  | |  |  |  | |  |  |  | | |  | |  | |  |
| neither | 1 | |  |  | 1 | |  |  | 1 | | |  | |  | |  |
| work exposure only | 1.66 | | 1.40 | 1.97 | 1.34 | | 0.91 | 1.97 | | | 2.10 | | 1.65 | | 2.68 |  |
| CMD only | 2.10 | | 1.81 | 2.44 | 3.84 | | 2.92 | 5.04 | | | 1.48 | | 1.15 | | 1.92 |  |
| both | 3.87 | | 3.26 | 4.60 | 5.00 | | 3.55 | 7.03 | | | 3.84 | | 2.97 | | 4.97 |  |
| Synergy index (S) | 1.63 | | 1.26 | 2.12 | 1.26 | | 0.85 | 1.87 | | | 1.79 | | 1.19 | | 2.70 |  |
| Physical workload/ CMD |  | |  |  |  | |  |  | | |  | |  | |  |  |
| neither | 1 | |  |  | 1 | |  |  | | | 1 | |  | |  |  |
| work exposure only | 2.34 | | 2.00 | 2.74 | 1.30 | | 0.90 | 1.89 | | | 3.43 | | 2.76 | | 4.26 |  |
| CMD only | 1.92 | | 1.62 | 2.26 | 3.41 | | 2.58 | 4.52 | | | 1.13 | | 0.82 | | 1.54 |  |
| both | 5.37 | | 4.56 | 6.31 | 5.86 | | 4.28 | 8.04 | | | 6.31 | | 4.95 | | 8.04 |  |
| Synergy index (S) | 1.94 | | 1.55 | 2.42 | 1.79 | | 1.20 | 2.66 | | | 2.08 | | 1.52 | | 2.84 |  |
| Computer work / CMD |  | |  |  |  | |  |  | | |  | |  | |  |  |
| neither | 1 | |  |  | 1 | |  |  | | | 1 | |  | |  |  |
| work exposure only | 0.99 | | 0.82 | 1.19 | 1.71 | | 1.20 | 2.43 | | | 0.73 | | 0.54 | | 1.00 |  |
| CMD only | 2.40 | | 2.09 | 2.76 | 4.34 | | 3.27 | 5.76 | | | 1.89 | | 1.53 | | 2.34 |  |
| both | 2.06 | | 1.70 | 2.50 | 4.84 | | 3.44 | 6.82 | | | 1.18 | | 0.84 | | 1.64 |  |
| Synergy index (S) | 0.76 | | 0.53 | 1.10 | 0.95 | | 0.65 | 1.38 | | | 0.28 | | 0.04 | | 2.03 |  |
| Shift work / CMD |  | |  |  |  | |  |  | | |  | |  | |  |  |
| neither | 1 | |  |  | 1 | |  |  | | | 1 | |  | |  |  |
| work exposure only | 1.77 | | 1.49 | 2.11 | 1.60 | | 1.09 | 2.35 | | | 1.92 | | 1.49 | | 2.47 |  |
| CMD only | 2.44 | | 2.12 | 2.81 | 4.40 | | 3.40 | 5.69 | | | 1.79 | | 1.44 | | 2.24 |  |
| both | 3.36 | | 2.73 | 4.15 | 4.25 | | 2.83 | 6.38 | | | 3.04 | | 2.20 | | 4.20 |  |
| Synergy index (S) | 1.07 | | 0.81 | 1.42 | 0.81 | | 0.51 | 1.29 | | | 1.19 | | 0.75 | | 1.91 |  |

**Supplemental Table 2.** Fully adjusted hazard ratios* (HR, 95% confidence intervals) for disability retirement by categories of the exposure variables.

|  | Any cause | | | Mental disorders | | | Musculoskeletal diseases | | | | | |
| --- | --- | --- | --- | --- | --- | --- | --- | --- | --- | --- | --- | --- |
| **Exposure** | HR | 95% CI | | HR | 95% CI | | HR | | 95% CI | | | |
| Hazardous exposure / CMD |  |  |  |  |  |  |  | |  | |  | |
| neither | 1 |  |  | 1 |  |  | 1 | |  | |  | |
| work exposure only | 1.42 | 1.19 | 1.68 | 1.28 | 0.86 | 1.89 | | 1.69 | | 1.32 | | 2.16 |
| CMD only | 2.15 | 1.85 | 2.49 | 3.64 | 2.76 | 4.79 | | 1.59 | | 1.23 | | 2.06 |
| both | 3.20 | 2.70 | 3.80 | 4.50 | 3.21 | 6.30 | | 3.06 | | 2.37 | | 3.95 |
| Physical workload/ CMD |  |  |  |  |  |  | |  | |  | |  |
| neither | 1 |  |  | 1 |  |  | | 1 | |  | |  |
| work exposure only | 1.89 | 1.61 | 2.23 | 1.26 | 0.85 | 1.87 | | 2.43 | | 1.93 | | 3.07 |
| CMD only | 1.95 | 1.65 | 2.31 | 3.20 | 2.40 | 4.25 | | 1.23 | | 0.90 | | 1.68 |
| both | 4.26 | 3.60 | 5.03 | 5.41 | 3.87 | 7.56 | | 4.46 | | 3.49 | | 5.71 |
| Computer work / CMD |  |  |  |  |  |  | |  | |  | |  |
| neither | 1 |  |  | 1 |  |  | | 1 | |  | |  |
| work exposure only | 1.22 | 1.01 | 1.48 | 1.87 | 1.30 | 2.68 | | 1.00 | | 0.73 | | 1.36 |
| CMD only | 2.36 | 2.06 | 2.72 | 4.13 | 3.11 | 5.47 | | 1.91 | | 1.55 | | 2.36 |
| both | 2.38 | 1.95 | 2.90 | 4.79 | 3.34 | 6.86 | | 1.53 | | 1.09 | | 2.14 |
| Shift work / CMD |  |  |  |  |  |  | |  | |  | |  |
| neither | 1 |  |  | 1 |  |  | | 1 | |  | |  |
| work exposure only | 1.40 | 1.17 | 1.68 | 1.41 | 0.97 | 2.07 | | 1.39 | | 1.07 | | 1.80 |
| CMD only | 2.43 | 2.11 | 2.80 | 4.12 | 3.18 | 5.34 | | 1.87 | | 1.50 | | 2.33 |
| both | 2.65 | 2.15 | 3.27 | 3.66 | 2.45 | 5.47 | | 2.23 | | 1.61 | | 3.09 |

* Models adjusted for gender, age, marital status, education, smoking, binge drinking, obesity, and chronic disease

**Supplemental Table 3.** Hazard ratios additionally adjusted for physical inactivity* (HR, 95% confidence intervals) for disability retirement by categories of the exposure variables.

|  | Any cause | | | Mental disorders | | | Musculoskeletal diseases | | | | | |
| --- | --- | --- | --- | --- | --- | --- | --- | --- | --- | --- | --- | --- |
| **Exposure** | HR | 95% CI | | HR | 95% CI | | HR | | 95% CI | | | |
| Hazardous exposure / CMD |  |  |  |  |  |  |  | |  | |  | |
| neither | 1 |  |  | 1 |  |  | 1 | |  | |  | |
| work exposure only | 1.46 | 1.22 | 1.75 | 1.40 | 0.93 | 2.10 | | 1.67 | | 1.29 | | 2.17 |
| CMD only | 2.18 | 1.86 | 2.56 | 3.91 | 2.91 | 5.24 | | 1.56 | | 1.19 | | 2.05 |
| both | 3.39 | 2.84 | 4.06 | 4.81 | 3.37 | 6.88 | | 3.31 | | 2.54 | | 4.31 |
| Physical workload/ CMD |  |  |  |  |  |  | |  | |  | |  |
| neither | 1 |  |  | 1 |  |  | | 1 | |  | |  |
| work exposure only | 1.95 | 1.64 | 2.32 | 1.46 | 0.97 | 2.20 | | 2.52 | | 1.97 | | 3.22 |
| CMD only | 2.01 | 1.68 | 2.39 | 3.41 | 2.51 | 4.63 | | 1.27 | | 0.91 | | 1.76 |
| both | 4.45 | 3.73 | 5.30 | 6.05 | 4.26 | 8.59 | | 4.72 | | 3.65 | | 6.11 |
| Computer work / CMD |  |  |  |  |  |  | |  | |  | |  |
| neither | 1 |  |  | 1 |  |  | | 1 | |  | |  |
| work exposure only | 1.16 | 0.95 | 1.43 | 1.82 | 1.24 | 2.68 | | 0.93 | | 0.67 | | 1.30 |
| CMD only | 2.40 | 2.08 | 2.77 | 4.20 | 3.13 | 5.65 | | 1.99 | | 1.60 | | 2.47 |
| both | 2.42 | 1.98 | 2.96 | 5.12 | 3.54 | 7.40 | | 1.48 | | 1.04 | | 2.11 |
| Shift work / CMD |  |  |  |  |  |  | |  | |  | |  |
| neither | 1 |  |  | 1 |  |  | | 1 | |  | |  |
| work exposure only | 1.43 | 1.18 | 1.72 | 1.39 | 0.94 | 2.06 | | 1.46 | | 1.12 | | 1.90 |
| CMD only | 2.49 | 2.15 | 2.89 | 4.31 | 3.27 | 5.67 | | 1.92 | | 1.52 | | 2.43 |
| both | 2.79 | 2.24 | 3.47 | 3.78 | 2.49 | 5.75 | | 2.47 | | 1.77 | | 3.45 |

* Models adjusted for gender, age, marital status, education, smoking, binge drinking, obesity, chronic disease and leisure-time physical inactivity

**
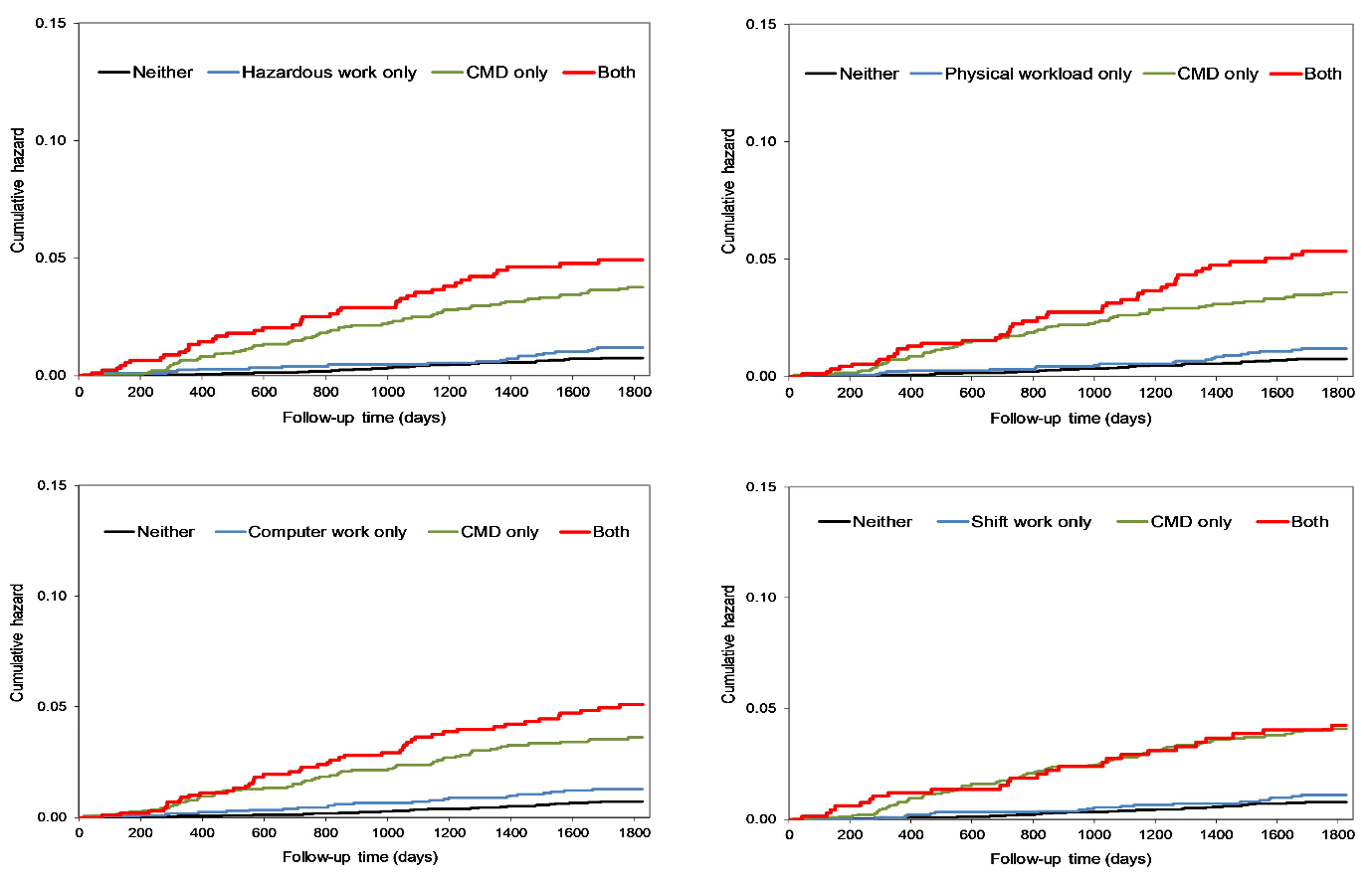
**

**Supplemental Figure 1**. Non-adjusted cumulative hazard of the occurrence of disability retirement due to mental disorders by joint exposure to physical work exposures and common mental disorders.


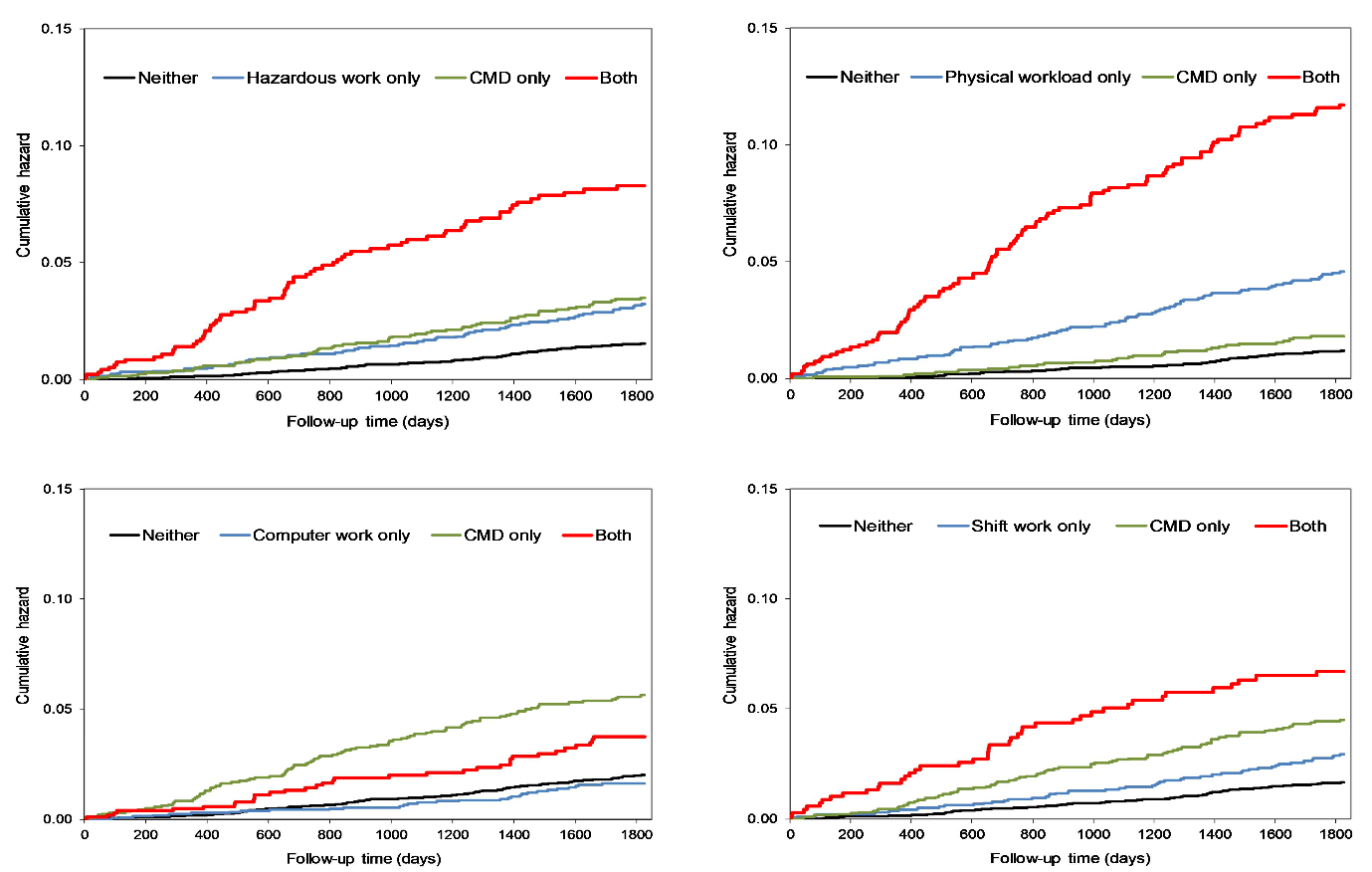


**Supplemental Figure 2**. Non-adjusted cumulative hazard of the occurrence of disability retirement due to musculoskeletal diseases by joint exposure to physical work exposures and common mental disorders.
